# From the unknown to spotlight: newly identified hormone adjusts hepatic cholesterol synthesis to dietary uptake

**DOI:** 10.1038/s41392-024-01882-5

**Published:** 2024-06-22

**Authors:** Yakum Bertrand Nkeh, Resul Gökberk Elgin, Gesine Saher

**Affiliations:** Project Group Biology of Lipids, Department of Neurogenetics, Max Planck Institute for Multidisciplinary Sciences, Göttingen, Germany

**Keywords:** Systems biology, Medical research

In a study published in *Cell*, Hu and colleagues discovered that the signaling activity of the intestinal hormone Cholesin leads to the attenuation of hepatic cholesterol synthesis in response to intestinal absorption thereby maintaining homeostatic levels of circulating cholesterol.^1^ These findings have major translational implications as they provide a potential new target for the management of hypercholesterolemia and associated disorders.

Cholesterol is an essential component of all mammalian cell membranes. As both its excess and its scarcity interfere with cell function, cholesterol metabolism is tightly regulated from the subcellular to the organism level.^2^ The clearance of cholesterol is achieved by conversion to bile acids followed by biliary excretion. The source of cellular cholesterol is either uptake or endogenous synthesis. Derivatives of certain intermediates of the cholesterol synthesis pathway have other important cellular functions, e.g., protein isoprenylation. Cholesterol synthesis is controlled transcriptionally and post-transcriptionally. Key to transcriptional control is the transcriptional regulator sterol responsive element binding protein-2 (SREBP-2). SREBP-2 activates transcription of several genes of cholesterol uptake (e.g., the low-density lipoprotein [LDL] receptor) and of the cholesterol synthesis pathway, including its rate-limiting enzyme hydroxy-methylglutaryl-coenzyme A reductase (HMG-CoA reductase). All nucleated cells can synthesize cholesterol but systemic cholesterol homeostasis in mammals is coordinated by the liver. During early postprandial phases, intestinal enterocytes distribute dietary cholesterol contained in chylomicron-type lipoprotein particles to lymphatics. After peripheral tissues absorbed lipids from these transport containers, chylomicron remnants reach the liver. Here, hepatocytes offset cholesterol influx (from dietary and reverse cholesterol transport sources) and cholesterol loss (due to lipoprotein release and conversion to bile acids) against hepatic synthesis to maintain circulating cholesterol levels. Notably, hepatic cholesterol production can account for up to 85% of the steady-state levels of plasma cholesterol. Although this feedback regulation has been known for decades, the mechanism by which the digestive tract and the liver communicate has remained enigmatic.

This elegant study by Hu et al.^1^ discovered the hormone function of the gene product of C7orf50 (humans) and 3110082I17Rik (mice), respectively, that share 64% amino acid identity. The authors termed this gene product Cholesin because its plasma levels raise within one hour after administration of dietary cholesterol following a 16-hour fasting. Similar effects were observed in mice, when cholesterol was administered directly or in the context of a high-cholesterol Western diet in comparison to a standard low-cholesterol chow. This finding argues in favor of a direct cholesterol effect with only minor influence of other lipids contained in high-fat high-sugar Western diets. Importantly, while these interventions immediately augmented intestinal cholesterol, they did not affect circulating plasma cholesterol levels within the first three hours of treatment. These findings were strikingly different in intestinal mutants of Cholesin; plasma Cholesin increased only slightly in response to dietary cholesterol but this dampened increase was paralleled by an acute 10–20% increase in plasma cholesterol levels. Although this hormone is expressed by several organs in mice, the intestine seems to be the major source of plasma Cholesin.

This prompted the researchers to analyze the mechanism by which dietary cholesterol stimulates the synthesis of intestinal Cholesin. Applying knockdown experiments in cell culture, mouse genetics (Niemann–Pick C1-Like 1 [NPC1L1] knockouts), and pharmacological inhibition of cholesterol uptake (Ezetimibe), they found that cholesterol uptake via the NPC1L1 transporter triggered Cholesin secretion from enterocytes. Again, despite these harsh interventions, plasma cholesterol levels remained stable. Cholesin circulated bound to exosomes, extracellular vesicles that are remarkably stable once released from cells and can therefore serve long-range inter-organ communication.

**Fig. 1:**
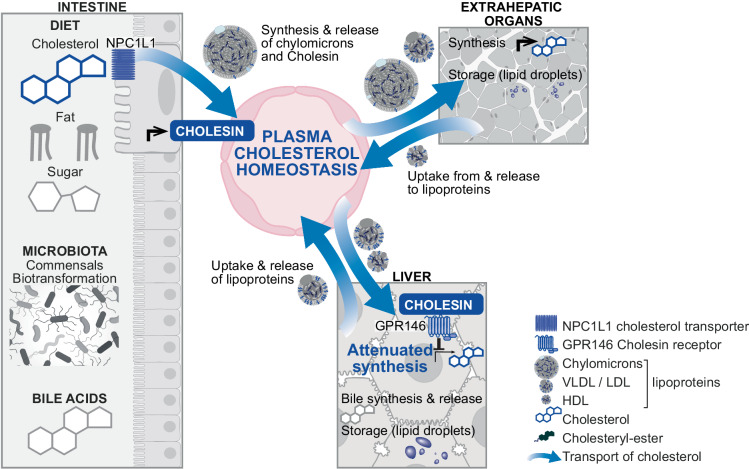
Regulation of systemic cholesterol homeostasis. The regulation of circulating cholesterol levels in mammals comprises a complex network of proteins that are involved in sensing, synthesis, uptake, storage in lipid droplets, secretion, and biochemical conversion of this lipid. Different regulatory layers enable tight control of steady-state cholesterol content at the cellular, organ, and organism level. To prevent postprandial hypercholesterolemia, the level of hepatic synthesis is adjusted to dietary absorption via the newly characterized hormone Cholesin. Triggered by cholesterol absorption via the Niemann–Pick C1-Like 1 (NPC1L1) cholesterol transporter, enterocytes express and secrete Cholesin. In hepatocytes, Cholesin binds its G protein-coupled receptor GPR146 followed by downstream inhibition of transcriptional activation of cholesterol synthesis. The transcriptional changes also affect uptake of low-density lipoproteins (LDL) and high-density lipoproteins (HDL) as well as the release of very low-density lipoproteins (VLDL). Figure adapted from images created with BioRender.com

Using a genome-wide clustered regularly interspaced short palindromic repeats (CRISPR) and CRISPR-associated protein 9 (Cas9) screen, the authors identified the orphan G-protein coupled receptor GPR146 on hepatocytes as a major factor of Cholesin-dependent maintenance of cholesterol homeostasis. In agreement with previous studies, upon Cholesin binding GPR146 downstream signaling attenuates the protein kinase-A (PKA)–extracellular signal-regulated kinases (ERK)-1/2 axis to SREBP2 transcription factor-mediated cholesterol synthesis in the liver. Hence, this excellent study provides evidence that under physiological conditions Cholesin serves the maintenance of plasma cholesterol homeostasis in mice despite dietary or pharmacological challenges (Fig. 1). As GPR146 is expressed in several extrahepatic organs, their role in systemic cholesterol regulation might prove an important perspective. The co-expression of Cholesin and GPR146 in the brain might hint to a local feedback regulation of cholesterol in the nervous system.

Importantly, the Cholesin-mediated feedback regulation of plasma cholesterol apparently also works in humans. Moreover, the single nucleotide polymorphism (SNP) rs1007765 in humans correlated with increased plasma Cholesin, a finding that could be replicated in human embryonic kidney cells. Moreover, when homozygous, this SNP was associated with low plasma cholesterol levels (total cholesterol and LDL-cholesterol), albeit still in the normal range. Future studies will bring this allele into context with the growing number of SNPs found to affect cholesterol metabolism.^3^

Given its regulatory role on hepatic cholesterol synthesis, could administered Cholesin dampen hypercholesterolemia? Repeated injections of Cholesin in mice reduced plasma cholesterol by 10–20%. In LDL receptor knockout mice fed a high-cholesterol Western diet, a model for hypercholesteremia, its application over several months led to a sustained amelioration of hypercholesteremia. An additive reduction to approximately 50% of plasma cholesterol levels was observed in a combination therapy with rosuvastatin. Statins are competitive inhibitors of HMG-CoA reductase, the rate-limiting enzyme of cholesterol synthesis. This treatment success is remarkable, especially in light of the per se reduced hepatic cholesterol synthesis rate in this mouse mutant.

Even though this study elegantly provides new mechanistic insight into cholesterol homeostasis, open questions need to be addressed by future studies: Cholesterol absorption is a complex process involving various factors that coordinates the import with the excretion of cholesterol and biliary sterols in enterocytes. Moreover, gut microbiota perform a number of biotransformations that can alter the pool of absorbable cholesterol. A recent study suggests that several bacterial strains differentially impact host cholesterol homeostasis and when in favorable commensals, plasma cholesterol levels.^4^ Cholesin is one of several hormones that influence cholesterol homeostasis. Elucidating the interplay between cholesin, estrogen, thyroid and glucagon might provide avenues to expand the fraction of plasma cholesterol that is susceptible to manipulation. The involvement of other nutrient sources such as sugar for the Cholesin-mediated cholesterol homeostasis remains to be investigated. Finally, elevated plasma cholesterol and LDL-cholesterol, respectively, are associated with cardiovascular disease but whether this relationship is causal is still a matter of debate. Rather, vascular inflammation appears to better predict future cardiovascular events,^5^ implying that a combination of anti-inflammatory treatment with Cholesin might provide therapeutic benefit.

Overall, this study impressively demonstrates how basic biochemical research in rodents and cell lines may pave the way to drug development for managing of a spectrum of metabolic diseases associated with elevated cholesterol.
